# 成人急性淋巴细胞白血病合并肺毛霉菌病1例报告并文献复习

**DOI:** 10.3760/cma.j.issn.0253-2727.2023.02.013

**Published:** 2023-02

**Authors:** 慧慧 樊, 文睿 杨, 馨 赵, 佑祯 熊, 康 周, 夏婉 杨, 建平 李, 蕾 叶, 洋 杨, 园 李, 莉 张, 丽萍 井, 凤奎 张

**Affiliations:** 中国医学科学院血液病医院（中国医学科学院血液学研究所），实验血液学国家重点实验室，国家血液系统疾病临床医学研究中心，细胞生态海河实验室，天津 300020 State Key Laboratory of Experimental Hematology, National Clinical Research Center for Blood Diseases, Haihe Laboratory of Cell Ecosystem, Institute of Hematology & Blood Diseases Hospital, Chinese Academy of Medical Sciences & Peking Union Medical College, Tianjin 300020, China

毛霉菌病是由毛霉目真菌引起的一种起病急、进展快、病死率高的侵袭性真菌感染，好发于糖尿病、血液系统恶性肿瘤、移植等免疫功能低下的患者[1]–[3]。近几年来随着血液科三唑类真菌预防治疗的普及，毛霉菌病发病率呈升高趋势，由于临床症状及影像学缺乏特异性，容易误诊或漏诊。我们报道1例成人急性B淋巴细胞白血病（B-ALL）患者诱导化疗期间发生肺毛霉菌病，应用两性霉素B胆固醇硫酸酯复合物（Amphotericin B Cholesteryl Sulfate Complex for Injection，ABCD）联合泊沙康唑（Pcz）针剂，并序贯外科手术治疗，感染控制得以继续化疗的病例。文献复习急性白血病合并毛霉菌病的相关报道，总结其临床特点及诊疗方案。

## 病例资料

患者，男，35岁，因“乏力2个月”于2021年8月12日入当地医院，血常规：WBC 1.6×10^9^/L，ANC 0.53×10^9^/L，HGB 69 g/L，PLT 31×10^9^/L。完善骨髓检查确诊B-ALL，应用VCLIP方案诱导化疗：长春地辛（VDS）4 mg第1、8、15、22天；环磷酰胺（CTX）1.3 g第1、15天；聚乙二醇门冬酰胺酶（PEG-asp）11 250 U第9、22天；去甲氧柔红霉素（IDA）15 mg第1～2天，10 mg第3、15、16天；泼尼松（Pred）70 mg第1～14天，40 mg 第15～28天。患者化疗后持续中性粒细胞减少/粒细胞缺乏（ANC<0.5×10^9^/L），间断发热，体温最高39.1 °C，伴咳嗽，少量咳痰，胸部CT示肺部感染。多次检测咽拭子、肛周拭子、血培养、痰培养均阴性，血清1, 3-β-D葡聚糖试验/半乳甘露聚糖检测（G/GM试验）阴性，结核杆菌抗体阴性。先后予头孢他啶、头孢哌酮舒巴坦钠、亚胺培南治疗1个月无效，后加用Pcz 300 mg/d口服1周无效停用，更换为伏立康唑针200 mg每12 h 1次，联合米卡芬净150 mg/d治疗1周余，患者一般状态恶化，为进一步诊治入我院。患者入院主诉发热伴咳嗽、咳痰、痰中带血、呼吸困难，不吸氧状态下血氧饱和度最低80％。查体：贫血貌，左肺呼吸音粗，右肺呼吸音低，余无明显异常。血常规：WBC 5.84×10^9^/L，ANC 5.45×10^9^/L，HGB 53 g/L，PLT 167×10^9^/L。生化：白蛋白21.3 g/L，丙氨酸转氨酶401.5 U/L，天门冬氨酸转氨酶57.4 U/L，直接胆红素45.6 µmol/L，间接胆红素37 µmol/L，血钾2.43 mmol/L，血钠133 mmol/L，余无异常。骨髓检查示ALL诱导化疗后完全缓解（CR）。胸部CT示：双肺感染、右肺大面积实变影、双侧胸腔积液（图1A）。入院行血培养、痰培养、拭子培养、G/GM试验均阴性，随即行支气管镜检查，镜下见右上肺叶坏死并右上气管分脊穿透。支气管镜检快速现场染色评估（ROSE）、支气管肺泡灌洗液（BALF）及痰宏基因组二代测序（mNGS）均提示微小根毛霉感染。予ABCD 150～200 mg/d联合Pcz针300 mg首日2次，后300 mg/d治疗。治疗2周复查生化指标恢复大致正常。患者间断发热，复查胸部CT显示双肺病灶较前缩小，但感染仍进展累及临近器官，逐渐出现气胸、支气管胸膜瘘、右侧第1～3肋骨骨质破坏、骨折断端进入肺组织、右肺脓肿（图1B～D）。脓肿液mNGS回报：微小根毛霉。抗毛霉菌治疗6周余患者一般状况改善，建议外科手术切除坏死病灶，家属因风险而犹豫不决。治疗9周患者改变体位时出现异物窒息，咳出一约5 cm×5 cm疑似坏死组织，紧急入住胸外科再次行支气管镜检查BALF、痰栓真菌涂片：均可见宽大透明飘带样无隔菌丝，符合毛霉菌形态。因感染累及右侧锁骨下动脉及静脉周围，行右侧锁骨下静脉支架置入术+右侧锁骨下动脉支架置入术+开胸探查+胸腔内粘连松解+心包内右全肺切除术+气管瘘肋间肌瓣修补术。术后送检肋骨真菌涂片及肺组织病理学，病理回报：慢性炎症性病变，可见小团游离坏死物中退变的真菌菌团，确诊肺毛霉菌病。术后患者体温恢复正常，胸部CT示：右肺切除术后，左肺感染性病变较前好转（图2）。术后8周复查骨髓示B-ALL复发。予抗毛霉菌药物治疗同时针对ALL行再诱导化疗。

**图1:**
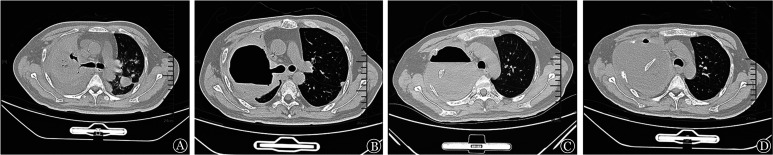
患者应用两性霉素B胆固醇硫酸酯复合物联合泊沙康唑治疗前后胸部CT变化 A 入我院前：双肺感染、右肺大面积实变影、磨玻璃及空洞影、双侧胸腔积液；B 药物治疗5周：两肺病灶较前缩小，但右侧肺内大量气体密度影，与右肺上叶支气管相通，右上叶支气管胸膜瘘；C、D 药物治疗6周、8周：右肺上叶结构消失，右侧胸腔内大量液体密度影，液平下第二肋骨断端疝入胸腔

**图2:**
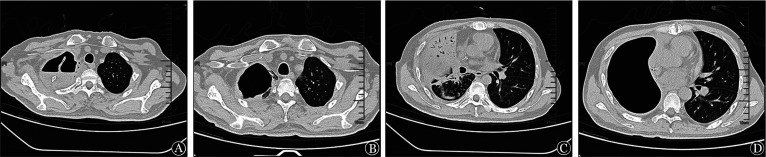
患者手术治疗前后胸部CT变化 A、C 右肺全切术前：右侧第一、二、三肋骨骨质破坏，肋骨断端疝入肺内；B、D 术后8周：右侧气体填充，左肺病灶明显减少

## 讨论及文献复习

本病例为急性白血病诱导化疗期间出现中性粒细胞减少伴肺部感染，血培养、痰培养、G/GM试验无明确病原菌提示。胸部CT表现并无特殊提示意义。早期治疗未能及时覆盖毛霉菌，感染进展迅速。住我院后经支气管镜检查，行ROSE、BALF/痰真菌涂片染色及mNGS检查为微小根毛霉感染。临床给予敏感药物治疗取得显著效果，外科得以干预，感染基本控制。本患者为我们已知的首例肺毛霉菌病导致支气管胸膜瘘及肋骨破坏并成功手术治疗的白血病病例。

急性白血病患者化疗后因中性粒细胞减少，免疫系统吞噬功能受损，是发生毛霉菌病的高危人群，我们复习相关文献，于万方数据库、PubMed数据库自建库至2022年4月的文献中以“急性白血病”、“毛霉菌病”、“acute leukemia”、“mucormycosis”为检索词检索出中文文献18篇，英文文献42篇。分析有详细病例资料、年龄≥14岁、组织病理学确诊符合条件的英文文献19篇，总结19例病例，其中急性髓系白血病（AML）11例，ALL 8例，结合本例，共纳入20例急性白血病合并毛霉菌病确诊病例进行毛霉菌感染临床特点、治疗方案总结（表1）。20例患者中，男13例，女7例，中位年龄51（14～82）岁。粒细胞缺乏17例（85％），其中14例（70％）发生于诱导化疗期间，2例（10％）发生于巩固化疗期间，1例（5％）发生于移植后。20例毛霉菌病类型：肺型12例（60％），播散型7例（35％），并均累及肺脏，牙周型1例。19例肺毛霉菌病患者中，13例行支气管镜检查，10例（76.9％）BALF或活检组织涂片染色阳性。20例患者中18例应用脂质体两性霉素B（L-AmB）为基础的抗毛霉菌治疗，16例（80％）感染好转或治愈患者中13例（65％）联合外科手术治疗；6例应用L-AmB和Pcz药物联合治疗，其中4例联合手术治疗，均预后良好。随访20例患者全因死亡9例（45％），其中6例（30％）死于感染，3例（15％）死于白血病复发，感染相关死亡患者中4例未行手术治疗，其中2例单用L-AmB，2例未应用药物治疗（表1）。毛霉菌目广泛存在于自然界，生长迅速，极具血管侵袭性，可引起广泛的组织梗死、局部侵犯和破坏。文献复习显示毛霉菌病好发于急性白血病诱导化疗的骨髓抑制期。毛霉菌病有六大临床表现型：鼻脑型、肺型、皮肤型、胃肠道型、播散型和少见类型[22]。不同宿主类型毛霉菌感染部位的倾向不同，血液系统恶性肿瘤以肺型为主，其次为播散型，且常累及肺部[23]。本次纳入的20例患者中播散型均累及肺脏，确诊肺毛霉菌病19例（95％）。

**表1:** 20例急性白血病合并毛霉菌病患者特点

例号	性别	年龄（岁）	白血病类型	治疗阶段	粒缺	感染部位	药物治疗	外科手术	临床类型	感染预后	转归	病例来源
1	女	47	AML	诱导	是	牙周	L-AmB	齿龈切除	牙周	治愈	治愈	2010美国[4]
2	男	52	AML	诱导	是	肺	L-AmB	右上肺叶切除	肺型	好转	未知	2013塞尔维亚[5]
3	男	44	AML	巩固	是	肺	L-AmB	无	肺型	死亡	死亡	2014日本[6]
4	男	82	AML	诱导	是	肺	无^a^	无	肺型	死亡	死亡	2014日本[6]
5	男	51	AML	诱导	是	肺	L-AmB	无	肺型	死亡	死亡	2016韩国[7]
6	男	59	AML	诱导	是	肺	L-AmB+Pcz	肋骨切除	肺型	好转	复发死亡	2016美国[8]
7	男	62	AML	诱导	是	肺/心脏	L-AmB	无	播散型	好转	未知	2019美国[9]
8	女	59	AML	诱导	是	肺	L-AmB	右上肺叶切除	肺型	治愈	复发死亡	2019日本[10]
9	女	53	AML	诱导	是	肺/皮肤	L-AmB+Pcz	无	播散型	治愈	治愈	2019美国[11]
10	女	64	AML	移植	否	肺/脑	无^a^	无	播散型	死亡	死亡	2019美国[12]
11	男	42	AML	巩固	是	肺/鼻脑/上颌	L-AmB	右肺切除	播散型	治愈	治愈	2020德国[13]
12	男	52	T-ALL	巩固	是	肺	L-AmB	右上肺叶切除	肺型	好转	好转	2001瑞典[14]
13	男	21	Ph^+^ALL	诱导	是	肺	L-AmB+Pcz	左肺上叶切除	肺型	治愈	治愈	2009西班牙[15]
14	男	15	T-ALL	诱导	是	肺	L-AmB	肺叶切除	肺型	好转	死亡	2016中国[16]
15	男	52	ALL	无	否	肺/皮肤	L-AmB+Pcz	无	播散型	好转	治愈	2020中国[17]
16	女	30	B-ALL	诱导	是	肺/肝脾肾	L-AmB	左上肺切除	播散型	好转	治愈	2020日本[18]
17	女	17	T-ALL	诱导	是	肺	L-AmB	局部肺叶切除	肺型	治愈	治愈	2020法国[19]
18	女	51	B-ALL	诱导	是	肺/胃肠	L-AmB+Pcz	空结肠部分切除	播散型	好转	死亡	2021中国[20]
19	男	14	Ph^+^ALL	巩固	否	肺	L-AmB	右下肺叶切除	肺型	好转	治愈	2022日本[21]
20（本例）	男	35	B-ALL	诱导	是	肺	L-AmB+Pcz	右肺全切	肺型	好转	复发死亡	2022中国

**注** AML：急性髓系白血病；ALL：急性淋巴细胞白血病；T-ALL：急性T淋巴细胞白血病；Ph^+^ALL：Ph染色体阳性急性淋巴细胞白血病；B-ALL：急性B淋巴细胞白血病；粒缺：粒细胞缺乏；L-AmB：脂质体两性霉素B；Pcz：泊沙康唑；^a^ 无毛霉菌敏感药

作为罕见的机会性感染，毛霉菌病在血液肿瘤患者中发病率不足2％，低于曲霉菌和念珠菌感染，但早期报道致死率高达75％～96％[23]，需引起临床重视。由于血液病诊治人群增加及三唑类抗真菌药物预防应用，毛霉菌病发病率呈升高趋势[24]，临床症状、体征、影像学无特异性，且因其嗜组织性及菌丝体较大难以入血，获取组织病理困难，组织培养往往阴性，早期诊断困难，常因延误治疗而导致死亡。

由于菌丝侵袭血管，导致组织坏死和血栓形成，毛霉菌病常进展迅速。影像学检查如胸部CT往往是临床诊治的首要依据。欧洲指南强烈建议高危患者每周进行CT检查[25]，有报道称94％的白血病患者在毛霉菌感染第1周行胸部CT检查可观察到反晕征[26]。胸腔积液和多发肺结节（>10个）是肺毛霉菌病常见的CT表现[5]，但无特异性。文献复习18例患者早期胸部CT显示多发肺结节，其中13例合并胸腔积液，5例伴反晕征。本病例胸部CT渐次出现胸腔积液、多发结节、反晕征等特点，符合肺毛霉菌病的影像变化特征。

目前尚无生物学标志物可识别毛霉菌，确诊依赖于病理组织中发现毛霉菌菌丝。相比外科手术，支气管镜检查临床可行性高，ROSE即时发现毛霉菌特异菌丝，即可识别毛霉菌感染，更宜早期进行，减少误诊及漏诊。支气管镜下活检病理阳性可确诊肺毛霉菌病。文献复习病例中行支气管镜荧光增白剂直接镜检和组织病理学涂片染色包括糖原染色或六胺银染色均发现：宽大无隔或少隔，飘带状菌丝（至少6～16 µm），多呈直角分枝，常伴血管闭塞，为毛霉菌的特点。

作为一项新型病原检测手段，mNGS通过对病原微生物的基因片段进行高通量测序来获得标本中的遗传信息，对于传统检测方法难以检测的病原微生物以及多种病原体混合感染具有更高的敏感性。mNGS检测病原体的核酸序列，在血浆中存活时间更长，受抗生素影响小。尤其BALF及痰液mNGS检查敏感快速，为毛霉菌病这类组织培养困难的感染提供高效临床诊断依据。本病例应用支气管镜检查联合mNGS检测首次识别出毛霉菌，对控制感染起到积极作用。

抗毛霉菌药物联合外科手术是毛霉菌病的主要治疗方案。由于早期鉴诊困难，对高危患者临床治疗尽早覆盖毛霉菌和曲霉菌至关重要。指南推荐足剂量L-AmB是治疗毛霉菌病最可靠的单药，可联合Pcz或艾沙康唑[25]。药物治疗剂量和疗程无统一标准，常用L-AmB剂量为5～10 mg·kg^−1^·d^−1^，Pcz首日300 mg 2次，后300 mg/d。因毛霉菌易侵蚀血管甚至骨质，单纯药物治疗常难以清除，无禁忌证者应尽快手术治疗。一项929例毛霉菌病回顾性临床研究表明，手术治疗是毛霉菌病良好疗效的独立预后因素[23]。患者无条件行外科手术时，更推荐药物联合治疗。本例患者双侧肺部毛霉菌感染应用两药联合治疗，一侧感染控制，另一侧病变范围缩小后，得以外科手术干预，为继续诊治白血病争取时机。文献复习并总结20例患者的治疗方案显示两种抗毛霉菌药物联合外科手术预后更好。但急性白血病合并毛霉菌病患者总体转归并不乐观，全因死亡率高，预后不良。

血液肿瘤患者合并毛霉菌病往往进展迅速，死亡率高，对于疑诊患者，积极联合呼吸科、外科、微生物室等进行多学科会诊协作，有助于早期诊断、改善预后。影像学检查、支气管镜检查、ROSE以及mNGS是快速诊断毛霉菌病的有效手段。尽早启动抗毛霉菌药物联合外科手术是治疗成功的关键，也是这类患者继续本病治疗的坚实基础。
